# Draft Genome Sequences of Three Novel *Clostridium* Isolates from Northern Iraq

**DOI:** 10.1128/genomeA.00033-16

**Published:** 2016-02-25

**Authors:** Srwa R. J. Rashid, Martha R. J. Clokie, Andrew D. Millard

**Affiliations:** a Department of Infection, Immunity and Inflammation, University of Leicester, Leicester, Leicestershire, United Kingdom; b Microbiology and Infection Unit, Warwick Medical School, University of Warwick, Warwick, United Kingdom

## Abstract

Three *Clostridium* sp. strains were isolated from soil and sediment collected from the Kurdistan region of Iraq. All three isolates were found to harbor putative prophages, with a CRISPR-Cas system found in strains C105KSO13 and C105KSO14.

## GENOME ANNOUNCEMENT

Three *Clostridium* sp. strains (C105KSO13, C105KSO14, and C105KSO15) were isolated from sediment and soil samples from the Kurdistan region of Iraq. Motility assays revealed that only C105KSO14 and C105KSE15 were motile. The strains were isolated by adding ~1 g of soil, with 10 ml of fastidious anaerobic broth (FA) (Oxoid, United Kingdom), supplemented with 250 µg·ml^−1^ cycloserine, 8 µg·ml^−1^ cefoxitin, and 0.1% sodium taurocholate. Cultures were incubated for 10 days in a MiniMACS anaerobic chamber at 37°C, prior to subculturing on Brazier’s cycloserine, cefoxitin, and egg yolk agar plates (Bio Connections, United Kingdom) for 48 h. Colonies were subcultured three times on BHI agar with 7% defibrinated horse blood. Bacterial genomic DNA was prepared from cultures by using a QIAGEN Genomic-tips kit according to the manufacturer’s instructions and sequenced using MiSeq (Illumina, USA). One nanogram of genomic DNA was prepared using the Nextera XT DNA sample preparation kit (Illumina) prior to sequencing on the MiSeq platform using the paired-end 2- × 250-bp (Version 2) protocol. Reads were trimmed with Sickle prior to assembly with Spades (1). The sequence was further checked for errors by mapping reads against the resulting assembly with BWA MEM using default settings, to correct any miscalled bases (2). Contigs with less than 5× coverage were removed from further analysis. All genomes had a minimum sequencing coverage of 25×. Putative prophages were identified using PHAST (3). Clustered regularly interspaced short palindromic repeat (CRISPR) arrays were predicted using PILER-CR (4) and putative spacer targets identified with CRISPRtarget against current plasmid and bacteriophage genomes (5). Genome annotation was carried out with Prokka1.11 (6).

Analysis of 40 single-copy phylogenetic makers with specI (7) did not enable us to classify these isolates to the species level. Analysis of the 16S rRNA suggests the closest relatives are *Clostridium oroticum* for C105KSO13, *Clostridium clostridioforme* for C105KSO14, and *Clostridium sphenoide* for C105KSO15. Isolate C105KSE15 has a genome size of 5.17 Mb, with 4,647 putative open reading frames (ORFs) and 55 tRNAs; C105KSE14 is 5.48 Mb, with 5,273 predicted ORFs and 57 tRNAs; and C105KSE13 is 3.21 Mb, with 2,982 predicted ORFs and 45 tRNAs.

All 3 isolates encoded between 1 (C105KSE15) and 12 putative prophage-like elements (C105KSE14). Strains C105KSO13 and C105KSO14 encoded a CRISPR-Cas system composed of two CRISPR arrays, with no putative CRISPR system in strain C105KSE15. The arrays in both strains contained multiple spacers ranging from 54 (C105KSO14) to 103 spacers (C105KSO13). Two spacers were found in C105KSO13, one was homologous to a sequence from a *Bacillus* phage MG-B1, with the second spacer homologous to sequences from *Staphylococcus* phages MR25 (8) and 88 (9). Three spacers in strain C105KSO14 were homologous to diverse plasmid genomes. The presence of multiple prophage elements and spacers to lytic phages highlights the evolutionary pressure bacteriophages exert on these bacteria and shows the importance of phages even in bacteria that are yet to be formally classified.

### Nucleotide sequence accession numbers.

The draft genome sequences of C105KSO13, C105KSO14, and C105KSO15 have been deposited in DDBJ/ENA/GenBank under the accession numbers, FBWL01000001, FBWO01000001, and FBWN01000001, respectively.
